# 

**Published:** 1842-04-30

**Authors:** 


**CONTENTS OF No. 18.**

Dr. Reynell Coates' Contributions to Surgery:
Note on the means of avoiding Excoriations of the Heel and Perineum in Fractures of the Lower Extremities, treated by Permanent Extension, (continued from page 262,) . . 273
Dr. Hartshorne's modifications of the simple buckskin gaiter, 273
The folded handkerchief, . . . . . . . 274
Dr. J. Rhea Barton's mode of employing the handkerchief, 275
BIBLIOGRAPHICAL NOTICE: The Pharmacopœia of the United States of America. By Authority of the National Medical Convention, held at Washington, A. D. 1840, . . . . . . 277
EDITORIAL: Progress of Hydropathy in Germany, . . . . . 280
Obituary: Dr. Carter N. Berkeley, of Philadelphia—Dr. Yellowly, of England, . . . . . . . . . 280
MISCELLANEOUS NOTICES: Mr. Bransby Cooper—The Astley Cooper Prize—Castleton Medical College, Vt. . . . . . 281
ANALECTA: Professor Stolz on Polypi of the Rectum in Children, . 281
M. Civiale on Catarrh of the Bladder, . . . . . 282
M. Velpeau's method of treating Varicose Veins, . . 282
Dr. Bartley's case of Paralysis of the Muscles of the Face, treated by Iodide of Potassium, . . . . . . 284
Dr. Clendinning's case of Hallucination and Epilepsy, . 285
M. Larrey on Aneurism of the Heart, . . . . . . 287
M. Donné on the composition of Urine during Pregnancy and Disease, . . . . . . . . . . . 287
M. Ricord on the treatment of Gonorrhœal Ophthalmia, . 287

